# Plasma exosomal miR-223 expression regulates inflammatory responses during cardiac surgery with cardiopulmonary bypass

**DOI:** 10.1038/s41598-017-09709-w

**Published:** 2017-09-07

**Authors:** Kin-Shing Poon, Kalaiselvi Palanisamy, Shih-Sheng Chang, Kuo-Ting Sun, Kuen-Bao Chen, Ping-Chun Li, Tso-Chou Lin, Chi-Yuan Li

**Affiliations:** 1 Department of Anesthesiology, China Medical University and Hospital, Taichung, Taiwan; 2 Graduate Institute of Clinical Medical Science, China Medical University, Taichung, Taiwan; 3 Division of Cardiology, Department of Medicine, China Medical University Hospital, Taichung, Taiwan; 4 Department of Pediatric Dentistry, China Medical University Hospital, Taichung, Taiwan; 5 School of Dentistry, China Medical University, Taichung, Taiwan; 6 Department of Surgery, China Medical University Hospital, Taichung, Taiwan; 7 Department of Anesthesiology, Tri-Service General Hospital, National Defense Medical Center, Taipei, Taiwan

## Abstract

Cardiopulmonary bypass (CPB) induces inflammatory responses, and effective endogenous homeostasis is important for preventing systemic inflammation. We assessed whether plasma exosomal microRNAs in patients undergoing cardiac surgery with CPB are involved in the regulation of inflammatory responses. Plasma samples were isolated from CPB patients (n = 21) at 5 specified time points: pre-surgery, pre-CPB and 2 hours (h), 4 h and 24 h after CPB began. Plasma TNF-α expression was increased after CPB began compared to that in the pre-surgery samples. Plasma IL-8 and IL-6 expression peaked at 4 h after CPB began but was downregulated at 24 h. The number of plasma exosomes collected at 2 h (55.1 ± 8.3%), 4 h (63.8 ± 10.1%) and 24 h (83.5 ± 3.72%) after CPB began was significantly increased compared to that in the pre-CPB samples (42.8 ± 0.11%). These exosomes had a predominantly parental cellular origin from RBCs and platelets. Additionally, the plasma exosomal miR-223 levels were significantly increased after CPB began compared to those in the pre-CPB samples. Further, exosomal miR-223 from plasma collected after CPB began downregulated IL-6 and NLRP3 expression in the monocytes. Here, we present the novel findings that increased plasma exosomal miR-223 expression during cardiac surgery with CPB might play homeostatic roles in downregulating inflammatory responses through intercellular communication.

## Introduction

Cardiac surgery with cardiopulmonary bypass (CPB) induces systemic inflammatory response syndrome (SIRS), which leads to substantial post-operative morbidity and mortality^1, 2^. CPB-induced inflammatory responses are activated by a myriad of events, including blood exposure to non-physiological surfaces, surgical trauma, ischaemic reperfusion and hypothermia^3^. Numerous immune cells, including neutrophils, monocytes and macrophages, participate in CPB-induced inflammation and produce both pro- and anti-inflammatory cytokines. Increased levels of TNF-α, IL-1, IL-6 and IL-8 mediate acute inflammatory response during CPB, and among them, TNF-α and IL-1 are identified as early mediators^4, 5^. In general, inflammatory activation is a protective surveillance mechanism; however, loss of control over inflammatory response results in systemic inflammation. Despite abundant knowledge on inflammatory mediators during CPB, the homeostatic regulatory mechanism of CPB remains elusive. To propose a better therapeutic strategy, understanding the regulatory mechanisms involved in inflammatory signalling during CPB are paramount.

microRNAs (miRNAs) are involved in developmental and pathological processes. miRNAs are non-coding RNAs (∼22 nt), which negatively regulate gene expression by post-transcriptionally targeting mRNA^6^. In certain clinical settings with SIRS characteristics, such as sepsis^7^, trauma^8^ and CPB^9, 10^, the deregulated expression levels of some miRNAs have been identified as early and sensitive biomarkers. Some miRNAs are involved in inflammatory signalling. miR-126 diminishes leukocyte trafficking and vascular inflammation by regulating endothelial VCAM-1 expression^11^. miR-155 targets NF-κB and negatively regulates oxLDL-induced atherosclerosis^12^. Let-7i-5p reduces cardiac inflammation and prevents fibrosis by downregulating IL-6 and collagen expression^13^. Emerging evidence shows miR-223 deregulation in patients with certain inflammatory and cardiovascular diseases^14–16^. miR-223 modulates haematopoietic lineage differentiation^17^ and plays a pivotal role in monocyte/macrophage differentiation by modulating the NF-κB pathway^18, 19^. Increased circulatory miR-223 in patients with rheumatoid arthritis mediates osteoclastogenesis via inflammatory activation^20, 21^.

Exosomes have emerged as cellular messengers in cell-cell communication. Stimuli and cell-type specific exosomes selectively package bioactive molecules (proteins, mRNAs and miRNAs) and modulate target gene expression and cellular function in recipient cells^22^. Exosomes regulate inflammatory responses via exosomal miRNAs^23^. We hypothesized that CPB-induced inflammatory responses are regulated by exosomal miRNAs, and this relationship has not yet been explored. Thus, the identification of deregulated exosomal miRNAs after CPB and their relationship with CPB-induced inflammatory mechanisms might be effective in preventing systemic inflammation.

The present study demonstrates the pivotal roles of plasma exosomes and exosomal miR-223 in downregulating IL-6 and NLRP3 expression through cellular communication with monocytes.

## Methods

### Patient and blood samples

Patients who underwent cardiac surgery with CPB from January 2010 to December 2014 (n = 21) were enrolled in the present study. This study complied with the Declaration of Helsinki, and the ethics committee of China Medical University approved the study protocol. Written informed consent was obtained from all the patients. Patients with rheumatoid arthritis, asthma, chronic bronchitis, cancer, and autoimmune disease as well as those receiving steroidal or nonsteroidal anti-inflammatory drug therapy were excluded from the study. At the beginning of the procedure, patients were pre-medicated with midazolam for arterial catheterization. Anaesthesia was induced with etomidate and maintained with sevoflurane containing oxygen, fentanyl, and rocuronium. All the patients were subjected to a routine median sternotomy and standard hypothermic CPB (Stockert S5 perfusion system, Sorin Group, Munich, Germany) with an extracorporeal membrane oxygenator (Medtronic Affinity Fusion Oxygenator, Medtronic Inc, Minneapolis, MN, USA). Porcine heparin was used at a dose of 300 U/kg as an anticoagulant (activated coagulation time > 480 sec) before and during CPB and then neutralized with protamine sulfate after CPB. Ten ml of blood was obtained from each patient via an arterial catheter at 5 pre-specified time points: after induction of anaesthesia (pre-surgery), before initiation of cardiac surgery with CPB (pre-CPB), and 2 h, 4 h and 24 h after cardiac surgery with CPB began. The blood samples were collected in ETDA tubes and centrifuged immediately at 1500 g for 12 minutes (mins) at room temperature. The isolated plasma was stored at −70 °C and used in further analyses. The plasma levels of TNF-α, IL-6 and IL-8 were determined using the Human ELISA Kit (Thermo Scientific, Rockford, USA) according to the manufacturer’s instructions.

### Plasma exosome isolation and size analysis

Plasma exosomes were isolated using a total exosome isolation protocol from a plasma kit (cat. no. 4484450, Invitrogen). Briefly, plasma samples were centrifuged at 2,000 g for 20 mins to remove cell debris. The supernatants were centrifuged again at 10,000 g for 20 mins, and the resultant pellets were discarded. The clarified plasma was diluted with PBS, and the mixtures were vortexed thoroughly. An exosome precipitation reagent was added, and the mixtures were incubated for 10 min. The samples were centrifuged at 10,000 g for 5 min, and the pellets containing exosomes were resuspended in PBS and used in future studies. The PBS-diluted exosomes were analysed for size using the dynamic light scattering (DLS) technique (Zetasizer Nano ZS, Malvern Instruments, UK).

### Characterization of the exosomal cellular origin using flow cytometry

To analyse cell-specific plasma exosomes, the isolated exosomes were resuspended in PBS containing 1% heat-inactivated foetal bovine serum (FACS buffer) and rested at 37 °C for 15 min. The exosomes were then incubated with 10 µg/ml of the appropriate primary antibody [CD235a (RBCs), CD40L (platelets), CD11b (monocytes)] plus the exosomal marker CD63. After incubation, the exosomes were washed twice with ice-cold FACS buffer and then stained with 0.25 µg of a FITC-linked secondary antibody for 30 min at 4 °C. To determine the total exosome percentage in the plasma samples, the isolated exosomes were labelled with CD63 and its respective fluorescence-conjugated secondary antibody. After washing with FACS buffer three times, the exosome counts were analysed by gating the live cell population based on FSC/SSC. Flow cytometry data were obtained by a FACS Canto flow cytometer using FACSDiva v8.0 software (BD Biosciences) and analysed using FlowJo v10.0.5 (Tree Star, Ashland, OR). The exosome counts were proportional to the number of fluorescently labelled exosomes, CD63 (total exosomes), [CD235a, CD63]-RBC-specific exosomes, [CD40L, CD63]-platelet-specific exosomes, and [CD11b, CD63]-monocyte-specific exosomes. The exosome counts in the pre-CPB samples were set to 100%, and the data are presented as the percentage of exosomes compared to the pre-CPB samples.

### Cell culture

THP-1 (ATCC-TIB-202), a human monocytic cell line, was purchased from American Type Culture Collection. The cells were grown in RPMI 1640 (Gibco, Invitrogen) supplemented with 10% foetal bovine serum (FBS) and 1% penicillin/streptomycin.

### Exosomal uptake analysis by flow cytometry and fluorescence microscopy

The PBS-diluted plasma exosomes (1:10) were treated with a primary CD63 antibody for 1 h at room temperature while shaking, which was followed by incubation with an APC-conjugated secondary antibody for 1 h at room temperature while shaking. The CD63-APC conjugated exosomes were then incubated with THP-1 cells for 1 h or 2 h and analysed for exosomal uptake by flow cytometry. Flow cytometry data were collected from a FACS Canto flow cytometer using FACSDiva v8.0 software (BD Biosciences) and analysed using FlowJo v10.0.5 (Tree Star, Ashland, OR). The data represent the percentage of exosomes taken up by THP-1 cells at various time points. For microscopic evaluation of exosomal uptake, THP-1 cells were treated with CD63-APC conjugated to either pre-CPB exosomes or exosomes collected 4 h after CPB began (Exo-4h-CPB) for 2 h, and the images were captured using a Zeiss fluorescence microscope.

### miRNA RT-qPCR for target gene validation

Plasma exosomal RNA was isolated using the miRNeasy mini kit (cat no. 74104, Qiagen, USA) following the manufacturer’s instructions. RNA concentrations were determined using a NanoDrop 2000 spectrophotometer (Thermo Scientific). A cell microRNA qPCR Array with QuantiMir™ (cat no. RA620A-1, System Biosciences) consisting of 39 haematopoiesis miRNAs (let-7a, let-7b, miR-10a, miR-10b, miR-16, miR-17-3p, miR-20a, miR-20b, miR-23a, miR-23b, miR-26a, miR-26b, miR-30b, miR-30d, miR-32, miR-33, miR-92, miR-93, miR-99a, miR-101, miR-107, miR-126, miR-130a, miR-142-5p, miR-3p, miR-146a, miR-146b, miR-155, miR-181a, miR-181b, miR-181c, miR-181d, miR-191, miR-193a, miR-193b, miR-197, miR-221, miR-223, miR-339) was used to identify differential plasma miRNA expression before and after CPB began. Real-time PCR was performed using a universal reverse primer, miRNA-specific forward primers, 2x master mix (Roche), and UPL probe #21 (Roche) in accordance with the manufacturer’s protocol (Applied Biosystems). Briefly, reverse transcription reactions were performed at 16 °C for 30 min, and the protocol consisted of 30 cycles of 30 °C for 30 sec, 42 °C for 30 sec, and 50 °C for 1 sec, followed by a 70 °C incubation for 5 min. RT-qPCR was performed on a real-time PCR system (Applied Biosystems) at 50 °C for 2 min, 95 °C for 10 min, and then 40 cycles of 95 °C for 15 sec and 60 °C for 60 sec. A list of various miRNA internal controls was selected from previous reports^24–27^ (sno95, miR-22, miR-92a, miR-423, miR-192, miR-451), and their expression levels in the plasma exosomes were analysed before and after CPB began. Among them, miR-92a was selected as the invariant miRNA, as it was stably expressed with no significant difference in the plasma exosomes before and after CPB began. The plasma miRNA expression levels were then calculated by normalizing them to those of miR-92a.

### Prediction of miRNA target genes

The TargetScan 6.2 (http://targetscan.org) database was used to predict the miRNA binding sites on the Hsa-IL-6 and Hsa-NLRP3 genes.

### miRNA and reporter vector construction

miRNA and reporter gene construction was performed as described previously^28, 29^. Genomic miR-223 precursor fragments were amplified by PCR using mouse genomic DNA as a template. The PCR products were cloned into the pLAS2-RFP vector at the NotI and XhoI restriction sites. After THP-1 cells were virally infected with the miRNAs, miRNA expression was detected using RT-qPCR. The miR-223 binding sites in the NLRP3 3′UTR sequence and the IL-6 coding sequence were cloned into the pmirGLO luciferase vector (Promega). Mutation constructs were obtained as described previously^30^. The mutant constructs of the NLRP3 3′UTR and IL-6 coding sequences were generated with a pair of primers containing the mutant sequences.

### Western blot analysis

Proteins were extracted from exosomes and THP-1 cells using RIPA lysis buffer, and their concentrations were determined using the Bio-Rad protein assay. Samples with equal protein concentrations were subjected to SDS-PAGE and then transferred to PVDF membranes (Perkin Elmer, Life Sciences). The blots were then blocked with 5% skim milk/TBS/Tween 20 for 1 h at room temperature and probed with primary antibodies against the target proteins (CD63, CD81, TSG101, IL-6, NLRP3) overnight at 4 °C. They were then washed with TBST and incubated with horseradish peroxidase-conjugated secondary antibodies (1:1000) for 1 h at room temperature. The blots were washed and incubated in ECL solution (Thermo Scientific, Waltham, MA) for 1 min and then exposed using the ImageQuant LAS4000 system (GE Healthcare).

### microRNA mimics and antagomir transfection

miRIDIAN miRNA mimics (Dharmacon) are single-stranded chemically enhanced oligonucleotides designed to mimic miRNA overexpression or knockdown. THP-1 cells were transfected with 100 nM of either the miR-223 mimics or the scramble mimics using the lipofectamine 2000 reagent (Invitrogen). After 24 h, the cells were plated for the luciferase reporter assay.

### Reporter assay

Cells (2 × 10^5^) were seeded in six-well plates and allowed to attach for 24 h. Co-transfection was then performed with either the miR-223 mimics and the NLRP3-3′UTR reporter vector or the miR-223 mimics and the IL-6-coding sequence vector using lipofectamine 2000 (Invitrogen). For the reaction mixture, 1 μg of the NLRP3-3′UTR vector (WT/MT), the IL-6-coding sequence vector (WT/MT) or the control vector and 100 nM of the miR-223 mimics, anti-miR-223 or the negative control (mimic/inhibitor) was added per well. Cell extracts were prepared 24 h after transfection, and luciferase activity was measured using the Dual-Luciferase Reporter Assay System (Promega).

### Statistical analyses

All the experiments were performed as 3 independent experiments in triplicate. The error bars represent the mean ± SD. Statistical analyses were performed using either generalized estimating equation (GEE) to assess the group, time, and group-by-time effects as well as to adjust the correlations arising from repeated measurements or ANOVA followed by Dunnett’s multiple comparisons test (Prism6, GraphPad Software Inc.). Significant p-values less than 0.05 are represented in the figures as *P < 0.05. The exact P-value is specified in the results section, while P-values less than 0.0001 are represented as P < 0.0001.

## Results

### Cardiac surgery with cardiopulmonary bypass increases plasma cytokine expression

**Figure 1:**
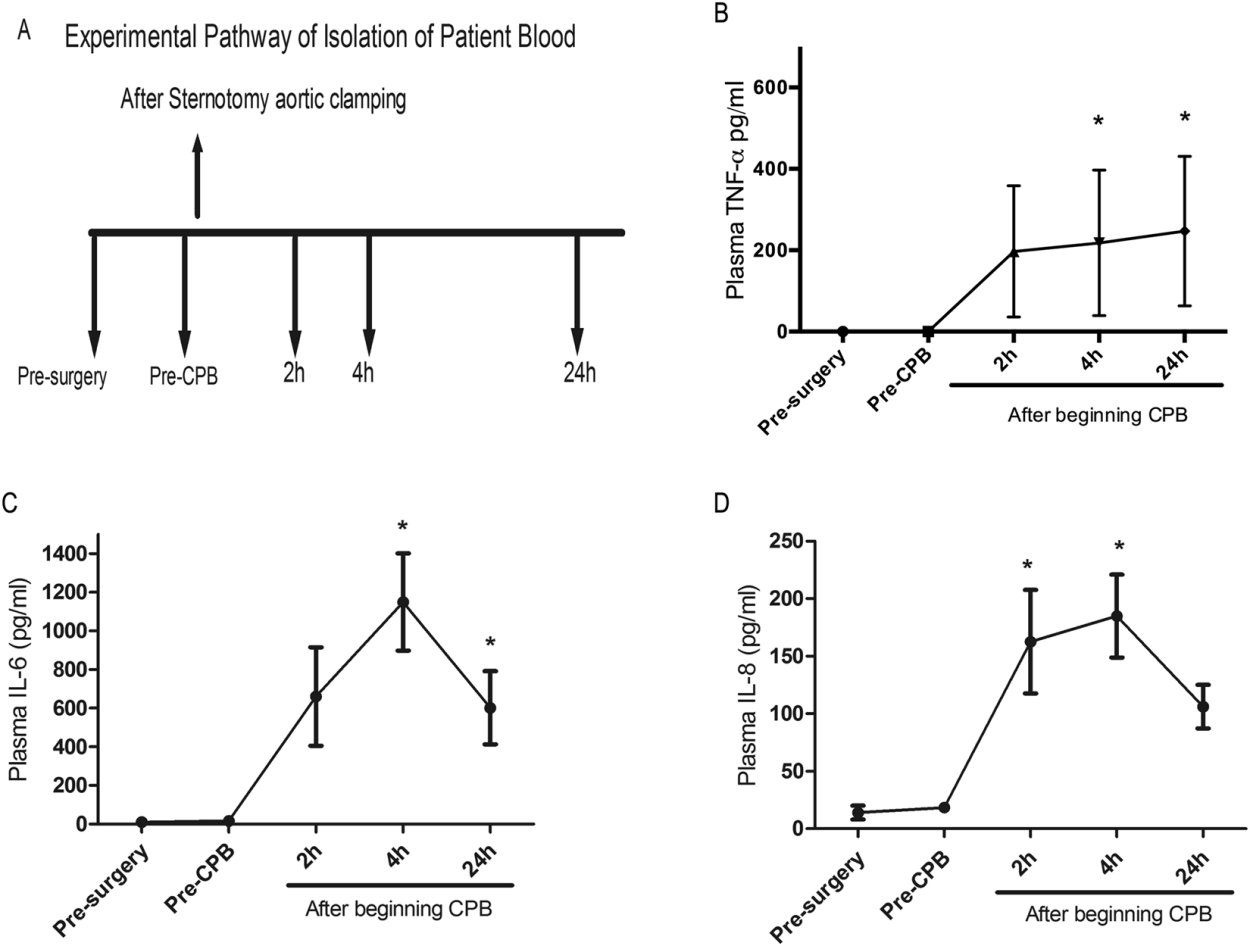
Increased plasma levels of TNF-α, IL-6 and IL-8 after CPB began. (**A**) Schematic representation of blood sample collection from CPB patients at different time points. (**B**–**D**) Plasma cytokine levels (TNF-α, IL-6 and IL-8) before and after CPB began (2 h, 4 h and 24 h) were quantified by ELISA. The results are expressed as the mean ± SD (n = 21). The experiments were performed as 3 independent experiments in triplicate. Statistical analysis was performed with the generalized estimating equation (GEE) (GraphPad Software Inc.). *P < 0.05 compared to the pre-surgery samples.

Table 1 describes the clinical characteristics of the CPB patients (n = 21), including their age, sex, EF%, CPB time (min), and cross-clamping time (min). The cardiac surgery procedures comprised coronary artery bypass grafts (CABG), ventricular septal defect (VSD) repairs, type A aortic dissections, mitral or aortic valve replacements, and atrial septum defect repairs with the endocardial cushion defect. Three patients had adverse post-operative (post-OP) events: two patients needed an intra-aortic balloon pump (IABP) and extracorporeal membrane oxygenation (ECMO) support for cardiogenic shock on post-OP day 1, and another patient suffered a cerebrovascular accident (CVA) on post-OP day 2. Figure 1A shows a schematic representation of the blood sample collection at different time points (pre-surgery, pre-CPB, and 2, 4 and 24 h after the cardiac surgery with CPB began). CPB induces systemic inflammatory responses by releasing cytokines^5^. To evaluate the inflammatory responses resulting from cardiac surgery with CPB, plasma TNF-α, IL-6 and IL-8 levels were determined. Figure 1B–D shows a significant increase in the plasma TNF-α, IL-6 and IL-8 levels in the samples collected after CPB began compared to those in the pre-surgery samples. TNF-α expression was increased after CPB began, with significant levels observed at 4 h (P < 0.05) and 24 h (P < 0.05). The IL-6 expression levels increased from 2 h, peaked at 4 h (P = 0.007) and then gradually decreased at 24 h (P = 0.001) after CPB began. Alternatively, the IL-8 levels were significantly increased 2 h after CPB began (P = 0.049), peaked at 4 h (P = 0.030), and subsided at 24 h. These results show that CPB mediates inflammatory responses by inducing cytokine expression.

**Table 1:** Clinical characteristics of the CPB patients.

Age (years)	49.8 ± 19.8
M/F	15/6
Ejection Fraction %	53.9 ± 14.0
Cardiopulmonary bypass time (mins)	127.8 ± 116.6
Cross-clamp time (mins)	65.4 ± 59.2

### Characterization of cell-type specific exosomal release in cardiac surgery with cardiopulmonary bypass

**Figure 2:**
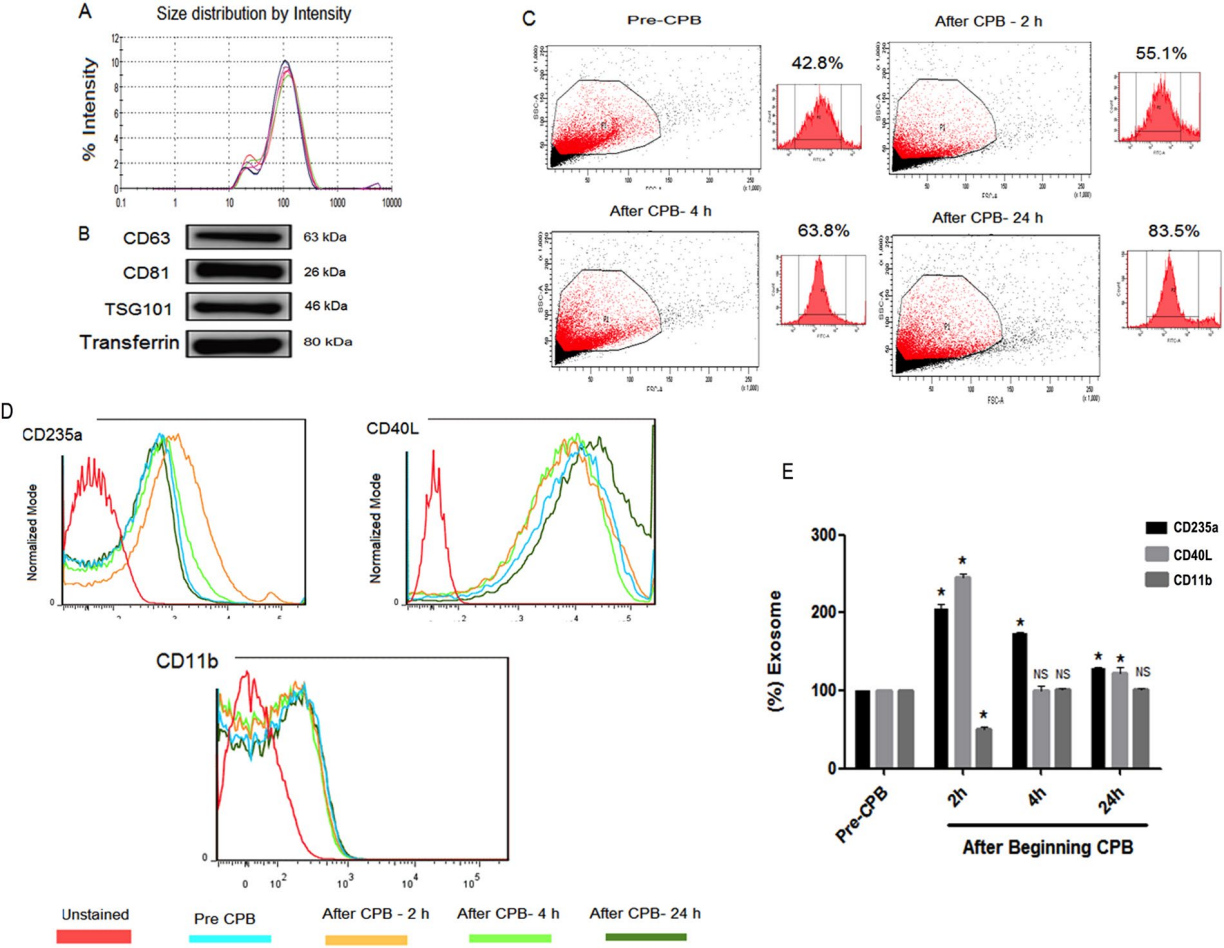
Plasma exosomal characterization in CPB patients. (**A**) Plasma exosome size distribution before and after CPB began (2 h, 4 h and 24 h), as determined by the dynamic light scattering technique. B. Plasma exosomal marker expression (CD81, CD63 and TSG101) was confirmed by western blot. (**C**) Plasma exosome counts were determined by analysing the FITC-conjugated surface marker expression (CD81) using flow cytometry (n = 15). (**D**) Flow cytometric analysis to characterize plasma exosomes from different cellular origins (RBCs-CD235a; platelets-CD40L; monocytes-CD11b) (n = 15). (**E**) Percentage of cell-specific plasma exosomes before and after CPB began, as determined by flow cytometry (n = 15). The results are expressed as the mean ± SD. Statistical analyses were performed with two-way ANOVA and post hoc Dunnett’s multiple comparisons using Prism6 (GraphPad Software Inc.). *P < 0.05 compared to the respective cell-specific markers in the pre-CPB samples. All the experiments were performed as 3 independent experiments in triplicate.

Plasma exosomes were analysed at various time points for their size distribution. Figure 2A shows that the exosomes had an average particle diameter size of 30–100 nm. Exosome identity was characterized by analysing the expression of exosomal surface markers (CD63, CD81 and TSG101) using western blot. Figure 2B shows exosomal marker expression in the pre-surgery samples. Additionally, we determined the exosome percentage before and after cardiac surgery with CPB through CD81 marker expression analysis by flow cytometry. An increased percentage of exosomes was observed in the samples collected at 2 h (55.1%), 4 h (63.8%) and 24 h (83.5%) after CPB began compared to the percentage in the pre-CPB samples (42.8%) (Fig. 2C). We further analysed cell-specific exosomal release by characterizing the respective cell surface antigens (CD235a, CD40L and CD11b) using flow cytometry (Fig. 2D). RBCs and platelets released a significantly higher percentage of exosomes after CPB began. RBC exosomes were found to be increased by 204% at 2 h after CPB began (P < 0.0001), 173% at 4 h (P < 0.0001), and 128% at 24 h (P < 0.0001), while platelet exosomes were increased 245% 2 h after CBP began (P < 0.0001), 100% at 4 h (NS), and 123% at 24 h (P < 0.0001) compared to the respective cell-specific markers in the pre-CPB samples (100%) (Fig. 2E). These results demonstrate that increased exosomal release from RBCs and platelets might be involved in regulatory events during CPB-induced inflammatory responses.

### Profiling haematopoiesis-related exosomal miRNAs in CPB patients

**Figure 3:**
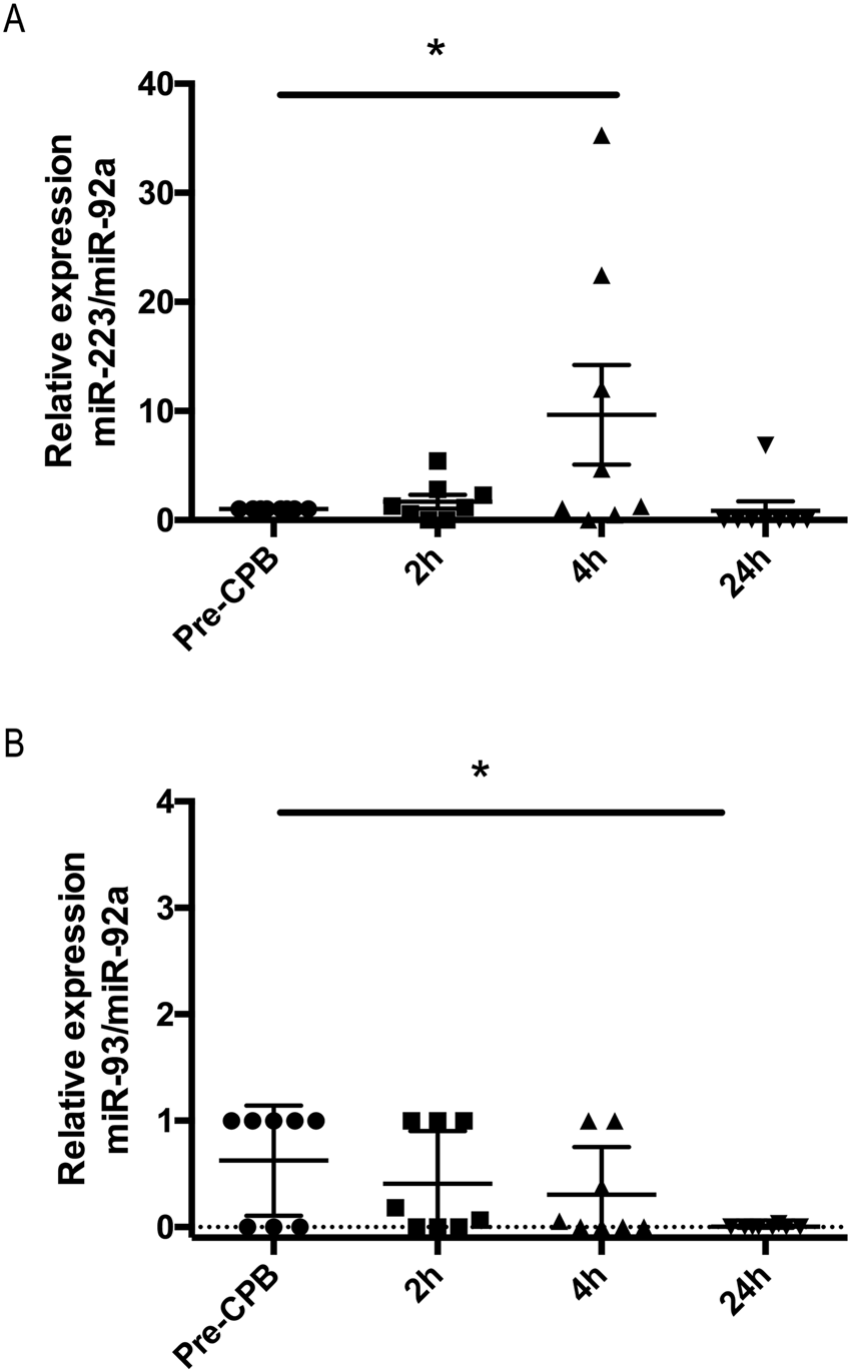
Differential plasma exosomal miRNA expression in CPB patients. (**A**,**B**) RT-qPCR analysis of the differential expression of haematopoiesis-related miRNAs (miR-223, miR-93) in plasma exosomes before and after CPB began (2 h, 4 h and 24 h). The results are expressed as the mean ± SD (n = 12). The experiments were performed as 3 independent experiments in triplicate. Statistical analysis was performed with one-way ANOVA and post hoc Dunnett’s multiple comparisons test using Prism6 (GraphPad Software Inc.). *P < 0.05 compared to the pre-CPB samples.

Since our study analyses the intercellular communication between haematopoietically derived cells, we analysed the differential expression of 39 haematopoiesis-related miRNAs (let-7a, let-7b, miR-10a, miR-10b, miR-16, miR-17-3p, miR-20a, miR-20b, miR-23a, miR-23b, miR-26a, miR-26b, miR-30b, miR-30d, miR-32, miR-33, miR-92, miR-93, miR-99a, miR-101, miR-107, miR-126, miR-130a, miR-142-5p, miR-3p, miR-146a, miR-146b, miR-155, miR-181a, miR-181b, miR-181c, miR-181d, miR-191, miR-193a, miR-193b, miR-197, miR-221, miR-223, and miR-339) in plasma exosomes using Roche UPL^®^ miRNA RT-qPCR. Among them, miR-223, miR-21a, miR-93, miR-20a, miR-16, Let-7b were detected in the plasma exosomes (data not shown). Additionally, two miRNAs (miR-223, miR-93) in samples harvested from different time points after CPB began showed significantly deregulated expression compared to that of the pre-CPB samples. miR-223 showed significantly increased expression at 4 h (P < 0.05), and miR-93 expression was significantly decreased at 24 h (P < 0.05) compared to the pre-CPB samples (Fig. 3A,B). These results imply that differentially expressed haematopoiesis-related miRNAs in plasma exosomes might play a regulatory role during cardiac surgery with CPB.

### Exosomal miR-223 downregulates IL-6 and NLRP3 expression in monocytes

miRNA target prediction using Target Scan-6.2 bioinformatics analysis showed that of the 2 miRNAs (miR-223 and miR-93), miR-223 had potential targets and a binding site for the IL-6 coding sequence and the NLRP3 3′UTR site. IL-6 is an important cytokine involved in CPB-induced inflammatory responses^31^. Linked to various cardiovascular diseases, NLRP3 inflammasomes function as pattern recognition receptors and mediate inflammatory responses by secreting proinflammatory cytokines^32, 33^. From differential miRNA expression and bioinformatics prediction analysis, we selected miR-223, which was significantly upregulated after CPB began, as the candidate miRNA for additional studies since it might have counterbalanced the inflammatory responses observed at 24 h. Monocytes regulate systemic inflammatory responses through cytokine production^34–38^, and TNF-α is a crucial regulator of CPB-induced inflammatory responses^39^. Thus, to determine the regulatory role of exosomal miRNAs against inflammatory responses, *in vitro* experiments were performed in a TNF-α-stimulated THP-1 monocyte cell line.

**Figure 4:**
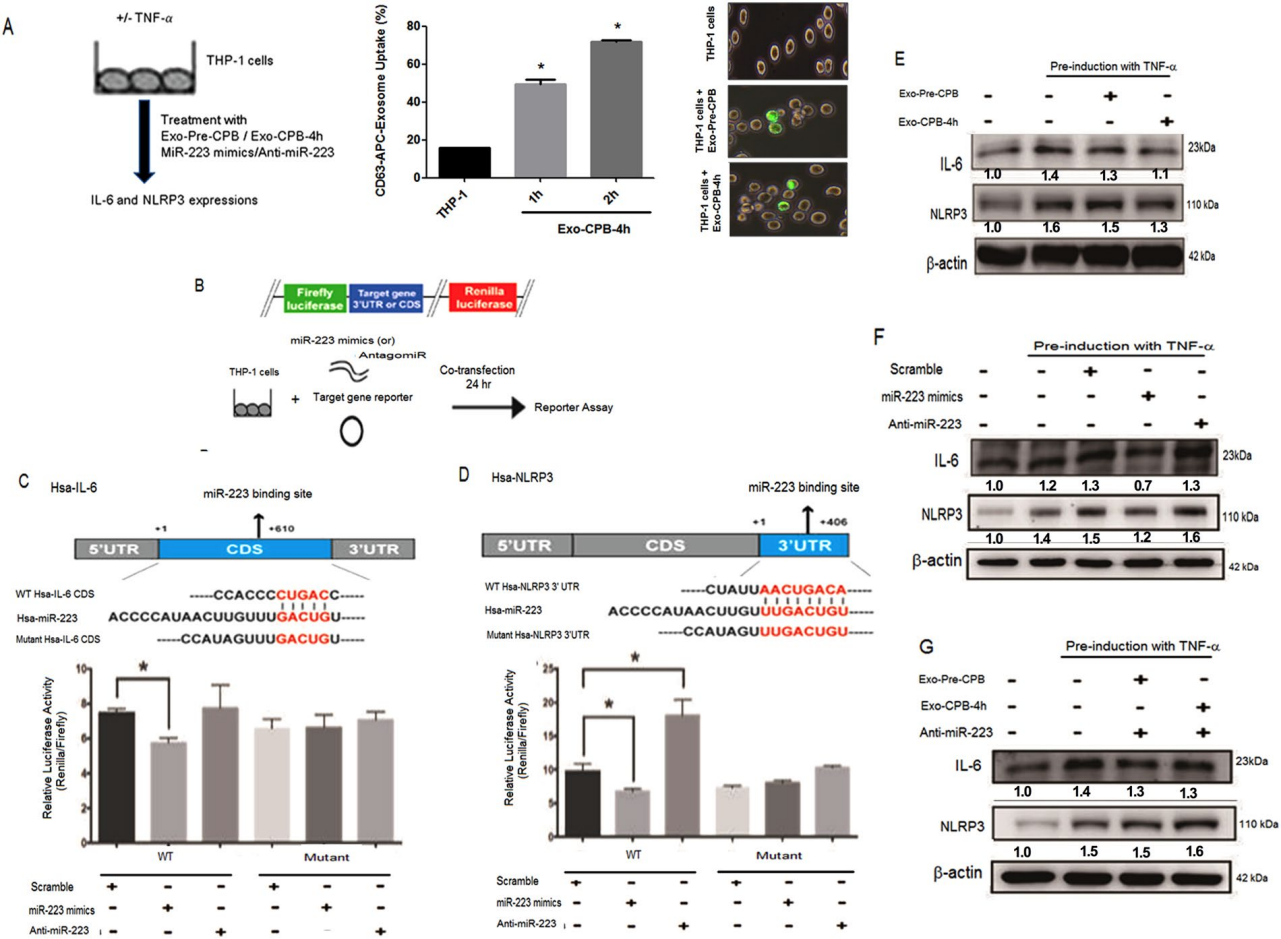
Exosomal miR-223 regulates IL-6 and NLRP3 expression in monocytes. (**A**) Schematic representation of plasma exosomes and miRNA (mimics/anti-miR) treatment with or without TNF-α-induced THP-1 cells. Exosomes conjugated with anti-CD63-APC were treated with THP-1 monocytes (for 1 h or 2 h) and analysed for the percentage of exosomal uptake by flow cytometry. The results are expressed as the mean ± SD. Statistical analyses was performed with one-way ANOVA and post hoc Dunnett’s multiple comparisons test using Prism6 (GraphPad Software Inc.). *P < 0.05 compared to the THP-1 cells. Fluorescence microscopy of CD63-APC-conjugated exosomes (green) (pre-CPB, Exo-CPB-4 h) and their uptake by THP-1 cells. Scale bar: 50 µm. (**B**) Schematic representation of the reporter vector (pmirGLO) carrying either the WT or mutant 3′UTR of the target gene or the CDS region. THP-1 cells were stimulated with TNF-α and cotransfected with either the WT or mutant 3′UTR of NLRP3 or the CDS of IL-6 and the scramble control, the miR-223 mimics or antagomiR for the reporter assay. (**C**) miR-223 binding sites for the IL-6 CDS as predicted by TargetScan. Luciferase assay using a reporter vector (pmirGLO) carrying either the WT or mutant CDS of IL-6 in THP-1 cells transfected with the scramble control microRNA mimics (controls, 100 nM), the miR-223 mimics (100 nM), the scramble control anti-microRNA inhibitor (controls, 100 nM) or anti-miR-223 (antagomir, 100 nM) for 24 h. (**D**) miR-223 binding sites for NLRP3 as predicted by TargetScan. Luciferase assay using a reporter vector (pmirGLO) carrying either the WT or mutant 3′ UTR of NLRP3 in THP-1 cells transfected with the scramble control microRNA mimics (controls, 100 nM), the miR-223 mimics (100 nM), the scramble control anti-microRNA inhibitor (controls, 100 nM) or anti-miR-223 (antagomir, 100 nM) for 24 h. (**E**) THP-1 cells induced with TNF-α were exposed to plasma exosomes isolated from pre-CPB and Exo-CPB-4h samples and analysed for IL-6 and NLRP3 expression. (**F**) TNF-α-induced monocytes were treated with miR-223 mimics or anti-miR-223, and IL-6 and NLRP3 expression was assessed. (**G**) Plasma exosomes from the pre-CPB and Exo-CPB-4 h samples were treated with anti-miR-223 and then exposed to TNF-α-induced monocytes to analyse IL-6 and NLRP3 expression. All the experiments were performed as 3 independent experiments in triplicate. The data are presented as the mean ± SD. *P < 0.05 compared to the WT scramble or control. One-way ANOVA followed by post hoc Dunnett’s multiple comparisons test was performed using Prism6 (GraphPad Software Inc.).

Figure 4A shows a schematic representation of exosome treatment with TNF-α-induced THP-1 cells. To evaluate the exosomal uptake of THP-1 cells, CD63-APC-conjugated exosomes were incubated with THP-1 cells. The flow cytometry results showed a time-dependent significant increase in exosomal uptake at 1 h (P = 0.0018) and 2 h (P = 0.0001) after CPB began. Additionally, the fluorescence microscopy results showed internalization of the CD63-APC-conjugated exosomes into THP-1 cells. To further validate the prediction, we cloned the 3′UTRs, the coding sequence and their seed sequence-mutated versions into downstream of the open reading frame (ORF) of a firefly luciferase reporter gene and assessed the ability of miR-223 to down-regulate luciferase expression (Fig. 4B). miR-223 bound to both the wild-type CDS of IL-6 (P = 0.04) and the wild-type 3′UTR of NLRP3 (P = 0.02) decreased the firefly luciferase expression compared to that of the WT scramble. Additionally, anti-miR-223 treatment with the wild-type 3′UTR of NLRP3 resulted in significantly increased luciferase expression (P < 0.0001) compared to that of the WT scramble. miR-223 did not bind the mutant CDS of IL-6 or the mutant 3′UTR of NLRP3 and showed nonsignificant levels of interaction (Fig. 4C,D).

We next sought to identify whether exosomes from patients who underwent cardiac surgery with CPB regulate inflammatory responses in monocytes. For this, patient exosomes isolated from pre-CPB samples and those collected 4 h after CPB began were treated with THP-1 cells that were pre-induced with TNF-α, and IL-6 and NLRP3 expression was determined. Figure 4E shows that the THP-1 cells induced with TNF-α exhibited increased IL-6 and NLRP3 expression compared to that of the control. Exosomes from samples collected 4 h after CPB began exhibited IL-6 and NLRP3 downregulation compared to that in the pre-CPB samples. Treatment with the miR-223 mimics resulted in downregulated IL-6 and NLRP3 expression in cells pre-induced with TNF-α. Additionally, anti-miR-223 did not downregulate IL-6 and NLRP3 expression (Fig. 4F). To confirm that exosomal miR-223 regulates IL-6 and NLRP3 expression, we treated the exosomes from pre-CPB samples and samples collected 4 h after CPB began with anti-miR-223 and assessed IL-6 and NLRP3 expression. Exosome treatment with anti-miR-223 did not downregulate TNF-α-induced IL-6 and NLRP3 expression (Fig. 4G). Together, these findings reveal that plasma exosomal miR-223 from CPB patients might communicate with monocytes to suppress IL-6 and NLRP3 expression and thereby regulate CPB-induced inflammatory responses.

## Discussion

Cardiopulmonary bypass (CPB) evokes a broad range of inflammatory responses during the intra- and post-operative periods. Here, we show the important role of exosomes and exosomal miRNA in cell-cell communication and their regulatory role in inflammatory responses during cardiac surgery with CPB. RBCs and platelets released predominant exosomes into circulation, and exosomal miR-223 that was significantly overexpressed after CPB began downregulated NLRP3 and IL-6 expression in monocytes.

Clinical outcomes after CPB depend on the degree of inflammatory activation. Blood circulation on artificial surfaces during CPB induces leukocyte activation and pro-inflammatory cytokine production^40, 41^. The observance of increased plasma TNF-α expression after CPB began agrees with results from previous studies on CPB-induced systemic inflammatory responses^4, 5, 39^. Furthermore, the IL-8 and IL-6 levels were significantly upregulated at 2 h and 4 h but declined 24 h after CPB began. Consistent data were previously published on CPB-induced inflammatory cytokine increases and their relevance to adverse clinical outcomes^2^. IL-8 levels have been strongly correlated with cardiac troponin-I levels and post-OP myocardial injury after CPB^42^. IL-6 participates in acute inflammation by inducing C3 and C-reactive protein expression^40^. Increased IL-6 expression during CPB is correlated with the severity of systemic inflammation and mortality^43, 44^. Thus, increased cytokine expression is involved in inflammatory responses in patients undergoing cardiac surgery with CPB.

Cell type-specific exosomes are secreted into the extracellular environment through a highly controlled process. Exosomes regulate both physiological and pathological responses and mediate diverse biological functions through intercellular communication^45^. Previously, Nieuwland *et al*.^46^ reported that the increased release of platelet microparticles from pericardial blood during CPB was associated with procoagulant properties *in vitro*. Here, we show for the first time that the increased number of plasma exosomes isolated from samples collected after CPB began were predominantly of cellular origin from RBCs and platelets. The elevated levels of exosomal release could be attributed to the CPB-induced effects on blood components. Blood contacts with extracorporeal devices and extravascular tissues during CPB are important factors of RBC lysis^46, 47^. The CPB-induced increase in plasma-free Hb levels is an early predictor of acute kidney injury (AKI)^48^, and changes in haemodynamic factors are strongly linked to AKI development^49^. CPB might be involved in platelet dysfunction and haemorrhage^50^, and platelet exosomes might cause endothelial cell damage and vascular dysfunction during sepsis^51^. Significantly higher levels of RBC-derived microparticles are associated with malaria infection^52^, and their cell-cell communication regulates parasite survival and differentiation^53^. Thus, increased exosomal release after CPB begins might have a functional significance in regulating CPB-induced inflammatory responses.

Circulating cell-free miRNAs are extremely stable and have emerged as disease biomarkers. Differentially expressed miRNAs under pathological conditions regulate cellular responses through post-transcriptional targeting^54^. CPB-induced systemic inflammatory responses are well studied, and a few studies have recently reported deregulated miRNA levels during CPB. Differential miRNA expression in the left ventricles of dogs from a canine CPB model of myocardial ischaemic reperfusion injury showed significantly downregulated miR-499 expression^10^. The research group lead by Zhang tested and identified cardiac-specific miR-1 as a urine and serum biomarker for acute myocardial infarction^55, 56^ and open-heart surgeries performed with CPB^9^. However, the functional significance of deregulated miRNAs remains elusive. We show that plasma exosomes harvested from patients undergoing cardiac surgery with CPB exhibited differentially expressed miRNAs. Importantly, exosomal miR-223 was upregulated 4 h after CPB began. miR-223 has been identified as a haematopoietic-specific miRNA with regulatory functions in differentiation and development^17^. The roles of miR-223 in monocyte/macrophage differentiation, RANKL-induced osteoclastogenesis, atherosclerosis, various cancers and metabolic disorders have been previously reviewed^57^. Hence, increased plasma exosomal miR-223 expression during cardiac surgery with CPB might play a regulatory role in CPB-induced inflammatory responses.

Exosomes and exosomal miRNAs are effective messengers in modulating target gene expression in recipient cells^58^. Here, we present novel findings on the communication between plasma exosomes collected 4 h after CPB began and monocytes, that led to the suppression of the inflammatory mediators IL-6 and NLRP3. Alternatively, the pre-CPB exosomes showed no downregulation of these inflammatory signalling molecules. Specifically, exosomal miR-223 from samples collected 4 h after CPB began showed suppression of these inflammatory mediators. Additionally, miR-223 directly bound to both the IL-6 coding sequence and the NLRP3 3′UTR site and repressed their expression. Elevated IL-6 expression during or after cardiac surgery with CPB is involved in CPB-induced inflammatory responses^31^. NLRP3 is a multimeric protein complex involved in cardiovascular pathogenesis^59, 60^. Activated by exogenous and endogenous factors, the NLRP3 inflammasome induces caspase-1-mediated proinflammatory cytokine (IL-1β and IL-18) maturation and secretion and induces pyroptosis via DAMPs. IL-1β and IL-18 are involved in the activation of other inflammatory cytokines^61, 62^. Differentially expressed miR-223 in the myeloid lineage regulates NLRP3 expression during the differentiation of monocytes to macrophages^63, 64^. Accordingly, upregulated exosomal miRNA-223 expression during cardiac surgery with CPB might be a critical regulator of CPB-induced inflammatory responses in monocytes.

Aside from the pharmacological approach to control CPB-induced inflammatory responses^65^, a more specific method for targeting proteins could be achieved through miRNA therapeutics^38^. Despite the reported changes in miRNA expression during CPB^9, 10, 49, 50^, their functional importance in the regulation of inflammatory homeostatic mechanisms is unknown. In this study, we demonstrate for the first time that the release of predominant exosomes from RBCs and platelets into circulation and miR-223 expression are significantly upregulated after CPB begins. Tight regulation of inflammatory responses by exosomal miRNA-223 occurs through the suppression of the IL-6 and NLRP3 inflammasomes in monocytes. This study shows that cellular interaction via exosomal communication and exosomal miR-223 expression might act as a critical regulator of CPB-induced inflammatory responses. Thus, deregulated plasma exosomal miR-223 expression might be identified as an early diagnostic marker correlated with morbidity during cardiac surgery with CPB.

## Electronic supplementary material

Supplementary Information
