# Supplementary material for: Plasma exosomal miR-223 expression regulates inflammatory responses during cardiac surgery with cardiopulmonary bypass
Source: Sci Rep. 2017 Sep 7;7:10807. doi: 10.1038/s41598-017-09709-w (PMC5589826; doi:10.1038/s41598-017-09709-w)
Supplement: Supplementary file 1 — Supplementary Information [file 41598_2017_9709_MOESM1_ESM.pdf]

Plasma exosomal miR-223 expression regulates inflammatory responses during cardiac surgery with cardiopulmonary bypass

Kin-Shing Poon[1,a] M.D., Kalaiselvi Palanisamy[2,a] Ph.D., Shih-Sheng Chang[2,3,a] M.D., Kuo-Ting Sun[4,5] M.D. Ph.D., Kuen-Bao Chen[1] M.D., Ping-Chun Li[6] M.D., Tso-Chou Lin[7,b] M.D., Chi-Yuan Li*[1,2,b] M.D.

Supplementary Data: Uncropped western blots

Figure 4E

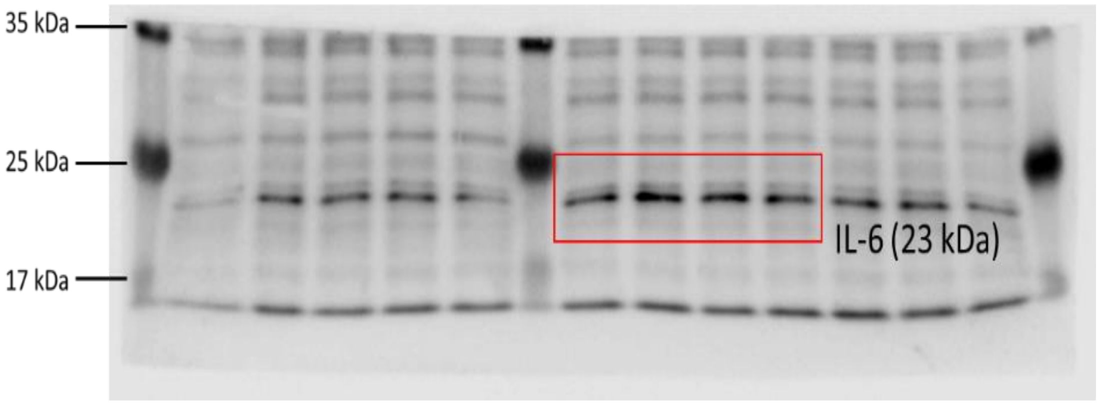

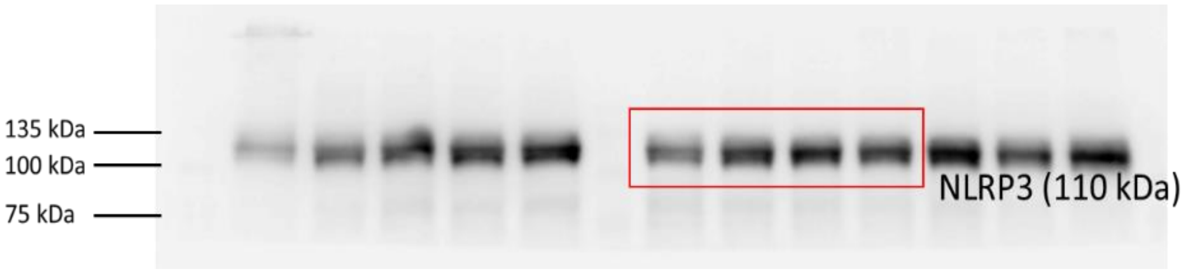

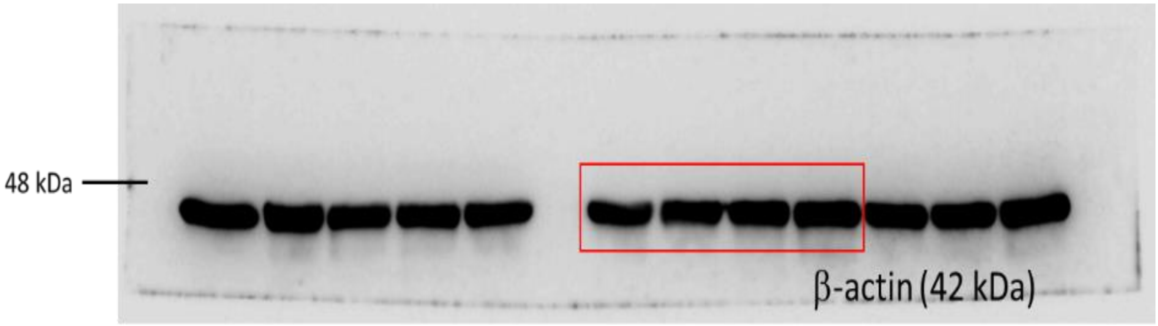

Figure 4F

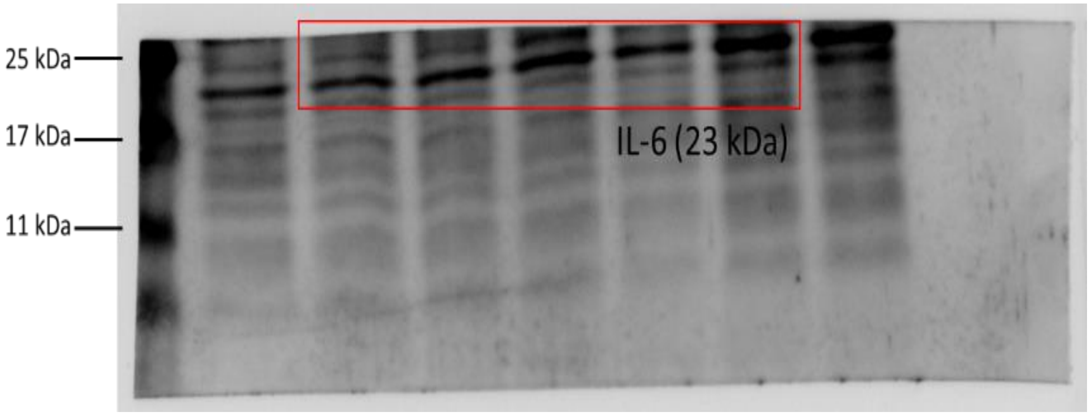
25 kDa
17 kDa
11 kDa
IL-6 (23 kDa)

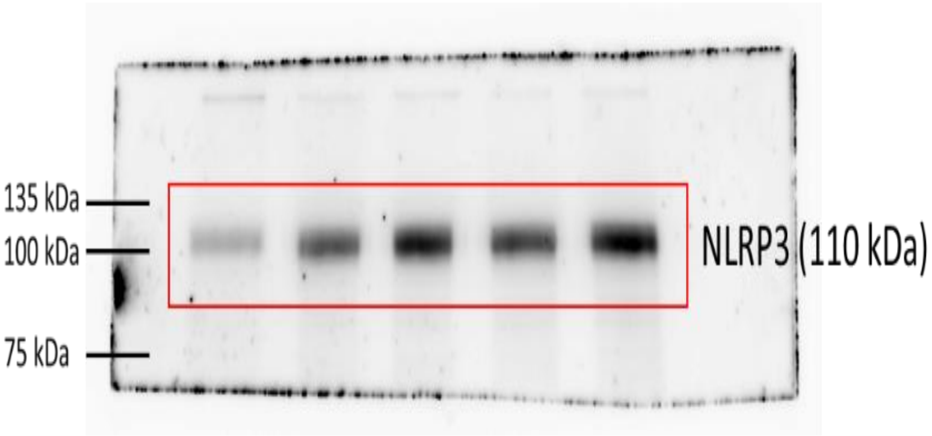
135 kDa
100 kDa
75 kDa
NLRP3 (110 kDa)

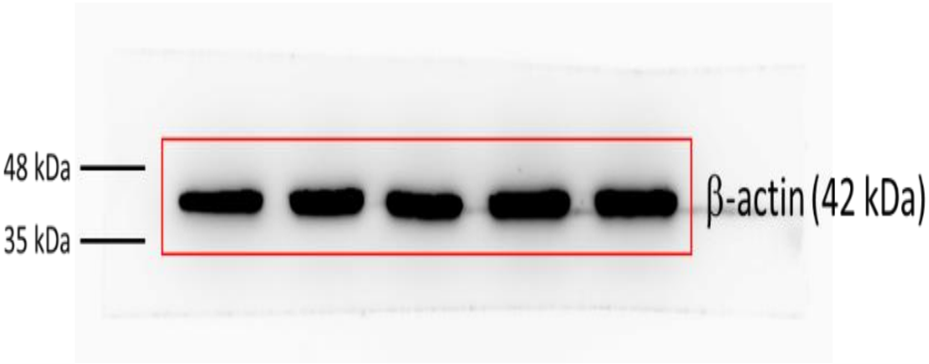
48 kDa
35 kDa
β-actin (42 kDa)

Figure 4G

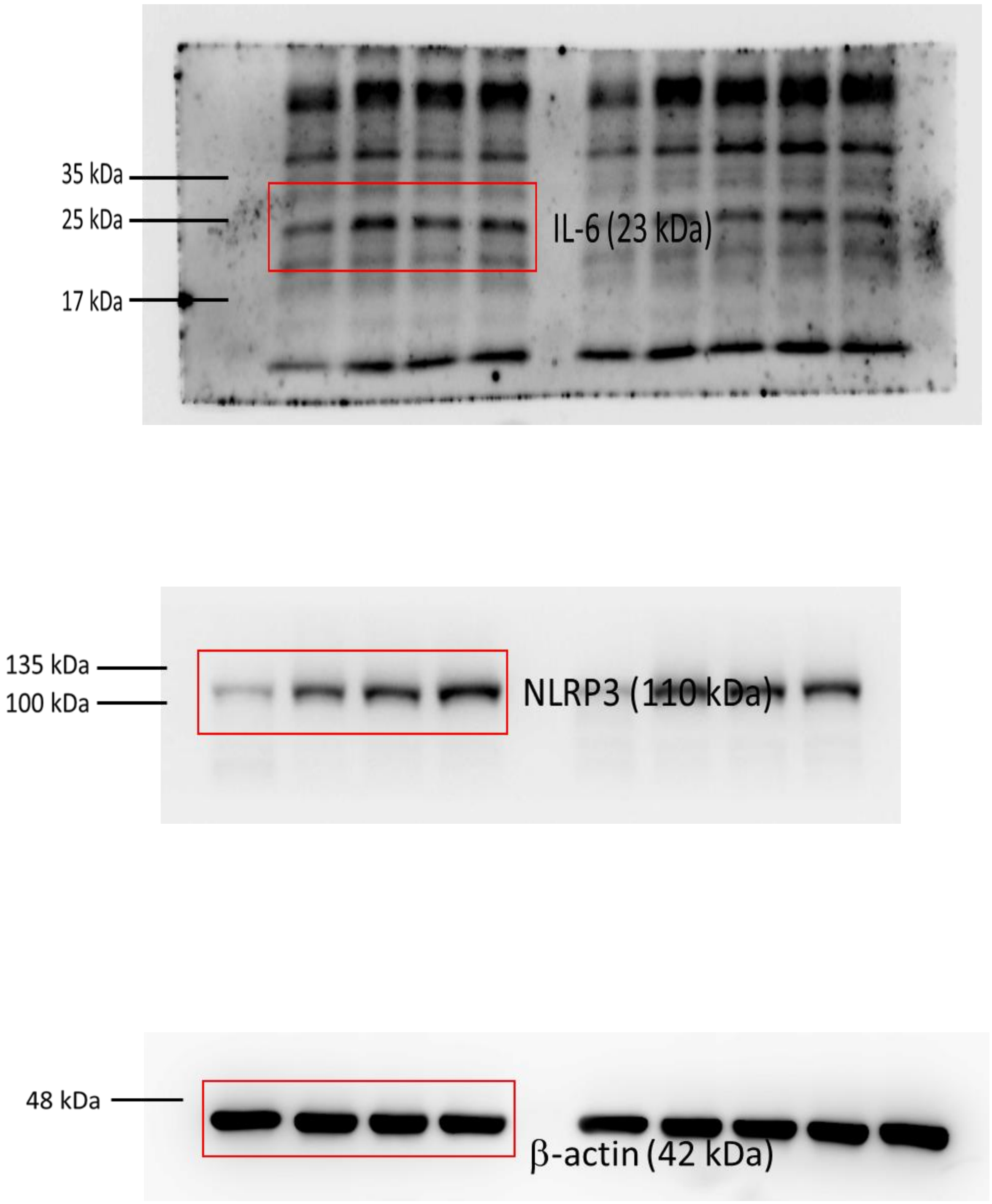
35 kDa
25 kDa
17 kDa
IL-6 (23 kDa)
135 kDa
100 kDa
NLRP3 (110 kDa)
48 kDa
β-actin (42 kDa)
